# Medical Bookkeeping

**Published:** 1914-05

**Authors:** 


### Medical Bookkeeping.

The *British Medical Journal* thinks that every physician should keep books, or should make his banker keep books for him. Unquestionably physicians are notably poor at keeping accounts and lose much by careless methods. Therefore the following suggestions from this excellent authority are worthy of consideration:

"Sir John Collie believes that every one who has any private income, however small, should keep two banking accounts at the same bank. The first, No. 1, is his ordinary current account, into which he should pay all the money he receives, whether he has been paid in cash or checks. It is an excellent rule never to spend a penny which has not come to one through his banking account. The physician should never get checks which have been paid to him cashed by the local tradesman. He should never pay his chauffeur with his midwifery fees. Every single item received from the physician's practice from one year's end to another should go through his banking account No. 1. If the physician has been tempted to use a five dollar bill which someone has paid him unexpectedly on a round, or if he has bought stamps with the money which some patient left at the office, then he should draw a check for the amount, cash it, and pay the money into his bank. Thus, and only thus, can he make his banker his bookkeeper.

"If the physician should die suddenly or wish to sell his practice, then, and then only, whether his other business books are kept well or not, the lawyer or the medical agency, or the purchaser who wades through his effects, will rejoice to find one method of knowing the gross receipts and getting some idea of the real intrinsic value of the business, and will from the physician's bank book alone tell the total cash receipts from the practice upon which to found the purchase price.

"At the end of every year the books should be balanced and if the doctor can not balance them himself, or has not the time, the comparatively small sum which a professional accountant will charge, say five or ten dollars, will be well repaid. If the physician is to keep the end ever in view before him, he must know definitely in black and white if his car, garden, or domestic expenses are increasing. If they are, then he will at least stop and think why this is so and whether he is getting the *quid pro quo*, whether indeed he can afford it, and above all, whether this increase in expenditure is preventing provision for a rainy day. In short, proper bookkeeping teaches one to live within one's means. It has also incidentally the advantage of helping one to render a proper return to the Income Tax Commissioners. The physician's annual balance will of necessity show the charges properly made against his business. He will thus protect himself against over-assessment by the Income Tax Commissioners. Surveyors of taxes are very apt to form extravagant opinions of professional men's incomes, and assess accordingly. It is very difficult to upset such assessment without proper books of account.

"The author assumes that the doctor under consideration is a successful man, but it is equally, if not more, important for the struggling practitioner, who, do what he can, is unable to make both ends meet, that he should be in a position to know positively and without any chance for self-deception whether it is worth his while to struggle on in the endeavor to make success of a failure that is absolutely certain to come sooner or later."

---

"Make the most of yourself, for that is all there is of you."—*Ralph Waldo Emerson.*

